# Effects on the FDG distribution by a high uptake of brown adipose tissue at PET examination

**DOI:** 10.1186/s13550-014-0072-0

**Published:** 2014-12-14

**Authors:** Henry Lindholm, Fredrik Brolin, Cathrine Jonsson, Hans Jacobsson

**Affiliations:** Department of Radiology, Karolinska University Hospital Solna, SE-171 76 Stockholm, Sweden; Department of Medical Physics, Karolinska University Hospital Solna, SE-171 76 Stockholm, Sweden

**Keywords:** Brown adipose tissue, FDG/PET, Radiotracer distribution, Reproducibility

## Abstract

**Background:**

At fluorodeoxyglucose/positron emission tomography (FDG/PET) examinations, a generally increased uptake of the skeletal muscles is sometimes encountered. As the tracer distribution constitutes a ‘zero-sum-game’, the uptake of lesions as well as of normal tissues is reduced in these patients. This has to be considered at calculation of standardised uptake values (SUVs), especially at longitudinal examinations in the same patient. In the current study, a possible similar influence on the FDG distribution by a high uptake of brown adipose tissue (BAT) was studied.

**Methods:**

Twelve patients with strongly increased BAT uptake were examined twice with a mean of 5 days (*study group*). In six of these patients, there was at least one pathological lesion with increased uptake. The BAT uptake was normalised at the second examination after pretreatment with propranolol. SUVs of the pathological lesions and of the liver, spleen, lung, blood, skeletal muscles, bone marrow, gluteal fat, abdomen and heart were assessed. In order to control the effects of propranolol on normal organs/tissues, which could interfere with the findings, 25 age and gender matched normal controls were also studied (*control subjects*).

**Results:**

In the *study group*, there was only a lower bone marrow uptake after propranolol administration. Comparing the *study group* with the *control subjects*, the bone marrow activity was higher at examination before propranolol treatment compared to the *control subjects*. There was also a higher uptake of the spleen in the *study group* before propranolol treatment compared to the *control subjects*. There were no differences between the *study group* after propranolol administration and the *control subjects*.

**Conclusions:**

The differences found are small and cannot be explained, why they could be random phenomena. Together with, there were no differences between the *study group* after propranolol administration and the *control subjects*; it is concluded that an effect on the FDG distribution in patients with a strong BAT uptake by can be disregarded in clinical praxis. This is important at longitudinal examinations of patients undergoing tailored tumour therapy and in contrast to examinations in patients with a generally increased uptake of the skeletal muscles which significantly affects the distribution of the radiopharmaceutical.

## Background

We have previously reported that the distribution of fluorodeoxyglucose (FDG) at positron emission tomography (PET)-examinations more or less constitutes a ‘zero-sum-game’ [1]. This was studied by comparing the tracer distribution in patients showing a high uptake of the skeletal muscles with the tracer distribution at repeat examination made a few days later after normalising the muscular uptake. This was achieved after instructing the patient to refrain from physical activity, together with diazepam pretreatment. Both the uptake of pathological lesions and of various normal tissues significantly increased after normalising the muscular activity. This fundamental mechanism has previously been little studied. Obviously, it has to be considered as it may influence the interpretation of single standardised uptake values (SUVs) as well as of longitudinal examinations in the same patient at therapy assessments.

Another phenomenon that could affect the FDG distribution in the same way and disguise a relevant SUV is a high uptake by brown adipose tissue (BAT) which sometimes is encountered [2,3]. Such a possible effect was studied in the current investigation which was performed in the same way as in our previous study [1]. Patients with a high BAT uptake, and being soon re-examined on clinical grounds after normalisation of the uptake by treatment of propranolol (β-blocker) prior to the tracer administration, were assessed in retrospect. Propranolol effectively reduces the FDG uptake of BAT [4,5]. In 12 consecutive such patients, SUVs of various normal tissues were compared between the two examinations. In order to control the effects of propranolol on normal organs/tissues, 25 age and gender matched normal controls were also studied (*control subjects*).

## Methods

### Patients

Both included patient groups were evaluated in retrospect. The *study group* constituted all patients with a strongly increased BAT uptake of FDG being re-examined on clinical grounds during the last 5 years after pretreatment with propranolol at which the BAT uptake was normalised. There were 12 patients (11 women and 1 man; mean age 35 ± 12 years). All patients were examined because of a known or suspected malignancy, in six patients this being malignant lymphoma. Six patients showed a normal finding at the examination. In the remaining individuals, one showed a single lesion with increased uptake, one individual showed two pathological lesions and in four individuals there were ≥ 3 lesions. Propranolol (40 to 80 mg) was administered orally 1 to 1.5 h prior to the administration of FDG at the recall examinations. There was a mean of 5 ± 3 days between the two examinations. At the first examination there was a mean of 72 ± 10 min between tracer administration and examination. At the second study there was a mean of 68 ± 10 min until examination. The blood glucose level was available at both examinations in 10 patients. At the first examination, the mean value was 5.7 ± 0.5 mmole/l. At the second examination this was 5.3 ± 0.5 mmole/l.

The *control subjects* were taken from our previously collected 500 clinical patients with a normal, or near normal FDG distribution [6]. It was not possible to establish controls paired with regard to age and gender of the study group. Instead, subjects forming a group with a similar gender and age distribution as the study group were consecutively picked out while going over our register. Twenty-five individuals (3 men and 22 women) with a normal blood glucose level (mean 5.1 ± 0.6 mmole/l) were included. The mean age was 35 ± 12 years. There was a mean of 59 ± 5 min between tracer administration and examination. The study was approved by the Regional ethics research committee (Regionala etikprövningsnämnden i Stockholm, Karolinska Institutet, SE-171 77 Stockholm, Sweden).

### PET/CT examination

B-glucose was measured immediately prior to administration of FDG using the same glucometer, HemoCue® Glucose 201^+^ (Hemocue AB, Ängelholm, Sweden). A Biograph 64 TruePoint TrueV PET/computer tomography (CT) scanner (Siemens Medical Solutions, Erlangen, Germany) was used. FDG (4 MBq/kg bw) was administered i.v. The scan usually included the mid-skull to the proximal thigh. Prior to examination with contrast medium, a low-dose CT was performed for attenuation and photon scatter correction. Directly thereafter, the PET examination was done, followed by a diagnostic (full-current) CT with or without contrast medium. At examinations without contrast medium, the full-dose CT was used for attenuation and scatter correction. The diagnostic CT examinations were performed with a tube tension of 120 kV, a pitch of 0.8, a slice thickness of 1.2 mm and a rotation speed of 0.5 s. The current was set to 160 mAs (reference), and dose modulation (CARE Dose4D) was applied. In the examinations done only for photon attenuation and scattering correction, CT was performed with a tube tension of 120 kV, a pitch of 0.8, a slice thickness of 1.2 mm, a rotation speed of 0.5 s and a current of 50 mAs. CT acquisitions were always done with the breath-holding technique at a mean inspiratory level. 3D PET acquisition was done for 3 min at each bed position during normal tidal breathing. The PET data were subsequently reconstructed with the manufacturer’s 2D-OSEM algorithm (four iterations and eight subsets) using a 5-mm Gaussian post-reconstruction filter. The image matrix size was 168 × 168 with a slice thickness of 5 mm. In addition to attenuation and scatter correction, all data were corrected for dead time and random coincidences.

### Image analysis

Activity quantification was done in the PET images using Siemens syngo MultiModality Workplace (syngo MMWP, VE36A). Volumes-of-Interest (VOIs) of different sizes and shapes adapted to the various organs/tissues were manually drawn by combining the information from the CT and PET acquisitions. In the *study group*, both examinations in the same patient were simultaneously evaluated using the commercial software to align the PET acquisitions and applying identical VOIs at the same location between the two studies.

In the *study group*, a large VOI was allocated on each side of the mid-line encompassing the BAT activity of the neck and the periclavicular regions in the consecutive coronal sections at the first examination. SUV_max_ of both VOIs was calculated. The identical evaluation was done at the second examination.

In the *study group*, as well as in the *control subjects*, SUV_mean_ of the liver was calculated by averaging the values of VOIs with a volume of 20 to 50 cm^3^ allocated at the centre of the right and left liver lobes, respectively. SUV_mean_ of the spleen was similarly averaged from three elliptical VOIs with a volume of 4 to 8 cm^3^ in various portions of the organ. SUV_mean_ of the lungs was calculated by averaging the values of parasagittal, ‘flat’ VOIs with a volume of 25 to 50 cm^3^ in each lung, covering both the upper and lower lobes. Blood SUV_mean_ was assessed by averaging the activity of five VOIs with a volume of 0.8 to 2 cm^3^ allocated in different portions of the lumen of the large mediastinal vessels. As they could not be discerned from the diffuse mediastinal FDG activity in most patients, allocation of these VOIs was done using the CT images. In cases of increased activity of the vessel wall, this was avoided. Muscular SUV_mean_ was calculated by averaging the values of elliptical VOIs allocated in the right and left shoulder muscles (25 to 50 cm^3^), in both psoas muscles (5 to 10 cm^3^) and in the right and left gluteal muscles (25 to 50 cm^3^), respectively. Bone marrow SUV_mean_ was calculated by averaging the values of elliptical VOIs with a volume of 2 to 5 cm^3^ allocated in both iliac crests and spherical VOIs with a volume of 1 to 1.5 cm^3^ in each lumbar vertebral body. If a vertebra was not possible to evaluate because of a compression or extensive spondylosis, it was excluded. In a few studies, one or two of the lower dorsal vertebrae were instead included. SUV_mean_ of the gluteal fat was assessed by allocating an elliptical VOI with a volume of 5 to 25 cm^3^. Abdominal SUV_mean_, mainly representing intestinal activity, was calculated from a ‘flat’ elliptical VOI with a volume of 200 to 500 cm^3^ allocated in the mid-abdomen avoiding the urinary tracts, female internal genital organs and any pathological lesion. The cardiac uptake was assessed by enclosing the left myocardium by a VOI from which SUV_mean_ and SUV_max_ were calculated.

SUV_max_ of the tumour/metastases were identically calculated at both examinations in the six patients of the *study group* showing pathological lesions. In the 2 individuals with 1 or 2 lesions, all were assessed, while in the 4 individuals with ≥3 lesions, 3 randomly selected lesions were assessed. In each of these patient, SUV_max_ was averaged. The values were thereafter averaged and the uptake before and after propranolol treatment compared.

### Propranolol effect on BAT

The BAT activity was normalised in all individuals between the two examinations at visual evaluation. This was an inclusion criterion. Mean SUV_max_ of the VOI encompassing the brown fat decreased at the right-hand side of the mid-line from 12.9 ± 7.1 to 1.7 ± 0.4 and on the left-hand side from 12.6 ± 7.5 to 1.7 ± 0.4, respectively. 1.7 is in the same range as the normal blood activity of the PET system used [6].

### Statistical methods

The distribution of the data allowed for the use of the paired Student’s *t* test for comparison between the two examinations of the *study group*. The Mann–Whitney *U* test was applied for comparisons between the *study group* and the *control subjects. P* < 0.05 was considered statistically significant.

## Results

The difference of SUV’s in the *study group*, i.e. between examinations in the same patient before and after pretreatment with propranolol, was very small. It was restricted to a slightly lower, but significant, bone marrow uptake after propranolol administration (Table 1).

**Table 1:** **Mean SUV’s (±SD) of various organs/tissues in 12 patients between FDG PET/CT examination**

**Organ/tissue**	**Before propranolol**	**After propranolol**	**Significance**
Liver SUV_mean_	2.1 ± 0.2	2.1 ± 0.3	ns
Spleen SUV_mean_	1.7 ± 0.2	1.7 ± 0.2	ns
Lung SUV_mean_	0.4 ± 0.1	0.4 ± 0.1	ns
Blood SUV_mean_	1.4 ± 0.2	1.4 ± 0.2	ns
Muscle SUV_mean_	0.8 ± 0.3	0.7 ± 0.2	ns
Bone marrow SUV_mean_	1.8 ± 0.3	1.6 ± 0.4	*
Gluteal fat SUV_mean_	0.2 ± 0.1	0.2 ± 0.1	ns
Abdomen SUV_mean_	1.1 ± 0.2	1.1 ± 0.3	ns
Heart SUV_mean_	3.4 ± 2.6	4.3 ± 3.2	ns
Heart SUV_max_	8.1 ± 6.3	10.6 ± 8.4	ns

FDG PET/CT examination was performed before and after pretreatment with propranolol.

Asterisk denotes 0.01 ≤ *p* < 0.05; ns, not significant.

The difference between the *study group* and the *control subjects* was also small. Also at this comparison, there was a difference of the bone marrow activity. This was higher at examination before propranolol pretreatment compared to the *control subjects* which showed no difference compared to examination after propranolol pretreatment (Table 2). In addition, there was a weak but significantly higher uptake of the spleen in the *study group* before propranolol treatment compared to the *control subjects*. There were no significant differences between the individuals of the *study group* after propranolol administration and the *control subjects*.

**Table 2:** **Mean SUV’s (±SD) of various organs/tissues in 12 patients and in 25 control (normal) subjects**

**Organ/tissue**	**Before propranolol**	**Significance**	**Control subjects**	**Significance**	**After propranolol**
Liver SUV_mean_	2.1 ± 0.2	ns	2.2 ± 0.4	ns	2.1 ± 0.3
Spleen SUV_mean_	1.7 ± 0.2	*	1.5 ± 0.3	ns	1.7 ± 0.2
Lung SUV_mean_	0.4 ± 0.1	ns	0.4 ± 0.1	ns	0.4 ± 0.1
Blood SUV_mean_	1.4 ± 0.2	ns	1.3 ± 0.3	ns	1.4 ± 0.2
Muscle SUV_mean_	0.8 ± 0.3	ns	0.7 ± 0.2	ns	0.7 ± 0.2
Bone marrow SUV_mean_	1.8 ± 0.3	*	1.5 ± 0.4	ns	1.6 ± 0.4
Gluteal fat SUV_mean_	0.2 ± 0.1	ns	0.2 ± 0.1	ns	0.2 ± 0.1
Abdomen SUV_mean_	1.1 ± 0.2	ns	1.1 ± 0.2	ns	1.1 ± 0.3
Heart SUV_mean_	3.4 ± 2.6	ns	4.3 ± 1.7	ns	4.3 ± 3.2
Heart SUV_max_	8.1 ± 6.3	ns	9.9 ± 4.4	ns	10.6 ± 8.4

FDG PET/CT examination was performed before and after pretreatment with propranolol.

Asterisk denotes 0.01 ≤ *p* < 0.05; ns, not significant.

In the six patients of the *study group* with pathological lesions, mean SUV_max_ of these increased from 6.5 ± 1.1 to 6.9 ± 1.1 between the two examinations. The difference was not statistically significant.

## Discussion

Only patients with a BAT uptake being normalised at the recall examination were studied. No individual with an incomplete effect by pretreatment of propranolol on the BAT activity was included. We tried to evaluate as many organs/tissues as possible. This could not be made of the brain as it was not included at many examinations. In addition, and unfortunately, it was not possible of the urinary tracts. Propranolol reduces the renal plasma flow and the glomerular filtration rate when given acutely [7]. Consequently, the β-blocker could exert an indirect effect on the FDG distribution. The renal parenchyma is too thin to calculate adequate SUVs, and because of normal variations in the diuresis, analysis of the urinary activity cannot be relevant.

The main finding of the study is that the difference between the examinations in the *study group* is very small and restricted to a minor difference of the bone marrow uptake. Surprisingly, and in contrast to our previous study, the heart uptake of FDG was not reduced but increased insignificantly after pretreatment with propranolol [5].

The administration of propranolol enabled the study but introduces an uncontrolled variable. Outside the effect on BAT, propranolol exerts pharmacodynamical effects on various organs/tissues which could interfere with the findings in *study group*. In an effort to control this, the *control subjects* were included in the study. Comparing the *study group* with the *control subjects*, the differences were also small. The higher bone marrow uptake of the *study group* before propranolol treatment compared to the *control subjects* cannot be explained. It upsets the observed difference of the bone marrow activity in the *study group* before and after propranolol administration, why this may be a random phenomenon. The higher uptake of the spleen in the *study group* before propranolol treatment compared to the *control subjects* can neither be explained and may have the same explanation. Even if the number of studied patients is small, the restricted effects together with that were no differences between the individuals of the *study group* after propranolol administration and the *control subjects* strongly indicates that an effect by propranolol can be disregarded.

## Conclusions

Along with the increasing availability of usually expensive drugs for tailored tumour therapy, treatment assessment constitutes a growing application of FDG/PET. Such longitudinal comparisons require strictly standardised conditions between the examinations which is difficult to achieve. The study indicates that in this respect differences in BAT-uptake between serial examinations can be disregarded. This is in contrast to a generally increased FDG uptake of skeletal muscles where the uptake of normal tissues, as well as of pathological lesions, is reduced. The difference may be explained by the much smaller tissue volume of BAT accumulating the tracer compared to the volume of the skeletal muscles.
